# Echocardiographic strategy for early detection of cardiotoxicity of doxorubicin: a prospective observational study

**DOI:** 10.1186/s40959-022-00143-0

**Published:** 2022-10-01

**Authors:** Diogo Pereira Santos Sampaio, João Batista Masson Silva, Daniela do Carmo Rassi, Aguinaldo F. Freitas, Salvador Rassi

**Affiliations:** 1 Division of Cardiology, Department of Internal Medicine, Faculty of Medicine, Federal University of Goiás, Rua 235 s/n, Setor Leste Universitário, Goiânia, Goiás 74605-050 Brazil; 2 Department of Echocardiography, Cardiovascular Division, Hospital das Clínicas of the Federal University of Goiás, Goiânia, Goiás Brazil

**Keywords:** Cardiotoxicity, Heart failure, Anthracyclines, Breast neoplasms, Echocardiography

## Abstract

**Background:**

Cancer chemotherapy using anthracyclines is associated with cardiotoxicity (CTX), and left ventricular ejection fraction (LVEF) analysis is not sensitive to early cardiotoxic changes. Left ventricular global longitudinal strain (LV GLS) monitoring helps screen subclinical CTX; however, the intervals at which it should be performed remain unclear. We aimed to evaluate the incidence of CTX in women with breast cancer and the associated factors and compare two echocardiographic monitoring strategies using two cutoff points for LV GLS variation.

**Methods:**

Patients with breast cancer prescribed doxorubicin underwent serial LVEF and LV GLS assessments using two-dimensional echocardiography every 3 weeks for 6 months.

**Results:**

We included 43 women; none developed a clinical CTX. Considering a relative reduction of LV GLS > 15%, subclinical CTX was present in 12 (27.9%) and six (14%) patients at 3-week and 3-month intervals, respectively (*P* = 0.28). Additionally, considering a reduction of > 12%, subclinical CTX was present in 17 (39.5%) and 10 (23.3%) patients (*P* = 0.16), respectively. There were no significant differences in either reference value at 3-week (*P* = 0.19) and 3-month intervals (*P* = 0.41). Age ≥ 60 years (*P* = 0.018) and hypertension (HTN) (*P* = 0.022) were associated with subclinical CTX in the univariate analysis.

**Conclusions:**

There was no difference in the incidence of subclinical CTX between the two cutoff points and no benefit in performing echocardiography every 3 weeks compared with quarterly monitoring. Advanced age and HTN were associated with the development of subclinical CTX.

## Introduction

The number of cancer patients undergoing chemotherapy has increased, which has enabled the identification of several adverse effects of antineoplastic medications, such as cardiotoxicity (CTX) [1]. Typical CTX is characterized by left ventricular dysfunction secondary to the use of anthracyclines, a common class of drugs used in the treatment of breast cancer, acute leukemia, and lymphoma [1–3]. This condition influences the prognosis of patients undergoing chemotherapy; hence, it can lead to delayed or interrupted treatment, with an unfavorable impact on the morbidity and mortality of cancer patients [4].

Anthracyclines increase the release of reactive oxygen species, change iron metabolism, and inhibit the enzyme topoisomerase 2ß, thus causing mitochondrial dysfunction and impairment of DNA repair, replication and transcription; therefore, culminating in cardiomyocyte death [2, 3]. Traditionally, CTX is monitored by the serial measurement of left ventricular ejection fraction (LVEF) using transthoracic echocardiography (TTE) [5–7]. However, this parameter is not very sensitive, and changes are detected only in a more advanced phase of CTX when myocardial damage has already occurred [8–10].

Early diagnosis of CTX at the subclinical stage is one of the main challenges for cardio-oncologists [11]; nevertheless, early cardioprotective therapy has been suggested to prevent and/or attenuate the deleterious effects of chemotherapy [9, 12–16]. Thus, serial TTE with myocardial deformation (strain) assessment using speckle tracking echocardiography has emerged as a useful method for CTX assessment in a preclinical phase. It is a safe, relatively simple, radiation-free method that helps to identify patients at higher risk for ventricular dysfunction. Reduced left ventricular global longitudinal strain (LV GLS) values are associated with the occurrence of CTX [8–10, 17, 18].

There is still no consensus regarding the optimal frequency of echocardiographic assessment in patients undergoing chemotherapy and the cutoff points for LV GLS reduction to detect CTX [10].. Therefore, this study aimed to evaluate CTX in women with breast cancer undergoing anthracycline treatment by analyzing the incidence of CTX; comparing two cutoff points for serial LV GLS assessment and two echocardiographic strategies to monitor CTX over the initial 6 months of treatment; and identifying clinical factors associated with the occurrence of CTX.

## Methods

### Study design and inclusion and exclusion criteria

This was an observational, longitudinal prospective study conducted among adult women with breast cancer without a history of previous cancer treatment who underwent treatment with doxorubicin at Hospital das Clínicas of the Federal University of Goiás (*Hospital das Clínicas da Universidade Federal de Goiás* - HC/UFG), Goiânia, GO, Brazil. The research project was approved by the Research Ethics Committee of the institution under the substantiated opinion n. 3,281,003, in accordance with the attributions defined in resolution 466/2012 of the Brazilian National Health Council. All participants provided written informed consent.

The inclusion criteria were: female sex, age ≥ 18 years, presence of breast cancer, no history of chemotherapy and radiotherapy and therapeutic planning for the use of doxorubicin. In contrast, the exclusion criteria were as follows: the presence of heart disease at baseline clinical evaluation (heart failure, coronary artery disease, congenital heart disease, cardiomyopathies, stage 3 hypertension (HTN) and clinically significant arrhythmias), echocardiographic changes (LVEF < 50%, moderately severe or severe valvular diseases, and segmental contractility changes) and inadequate acoustic window for TTE.

### Study procedures

The data were collected from April 2019 to June 2021. The patients’ demographic, clinical, and physical examination data were collected at the initial appointment. Eligible patients were referred for baseline TTE. The study protocol consisted of TTE complemented with LV GLS assessment before starting chemotherapy (baseline) and at 3-week intervals, coinciding with the cycles of doxorubicin administration (60 mg/m^2^/cycle). The tests were preferably performed within 48 h after chemotherapy. Each patient underwent a maximum of nine tests over the 6-month follow-up period.

TTE was performed at the department of echocardiography of HC/UFG by an experienced professional using a Philips CX50, Affiniti, or Epic CVx (Philips, Amsterdam, The Netherlands) device equipped with a 2.5–3.5 MHz multifrequency transducer. The images were obtained in multiple planes according to criteria established by the American Society of Echocardiography (ASE) [19]. All available conventional echocardiographic modalities were used to analyze structural and functional cardiac changes. All measurements and the echocardiographic evaluation of the chambers followed the recommendations of the ASE and the European Association of Cardiovascular Imaging (EACVI) [20].

LVEF was calculated using the modified Simpson’s rule. The endocardial border of the left ventricle was traced in apical two- and four-chamber planes at the end of diastole and systole to estimate cavity volumes. The left ventricle was divided into several disks along its longitudinal axis using these tracings, and the final volume was obtained by adding the volumes of the disks. LVEF was obtained by calculating the ratio between ejected (difference between diastolic and systolic volumes) and diastolic volumes [20].

LV GLS images were acquired in apical two-, three-, and four-chamber planes with the optimal electrocardiographic signal amplitude at 40–70 frames/s. The images of three consecutive cardiac cycle beats were digitally stored and analyzed using the Philips Qlab13® automated Cardiac Motion Quantification (aCMQ) software (Philips, Amsterdam, The Netherlands). LV GLS was measured according to consensus recommendations of the EACVI, ASE, and industry representatives [21].

Clinical CTX was defined as an LVEF reduction of 10% or more to values below 50% at any time during the assessment compared with baseline echocardiogram values. Subclinical CTX was defined in two ways: (1) a relative decrease of LV GLS greater than 15% at any time of the assessment and (2) a relative decrease of LV GLS greater than 12% at any time of the assessment, both compared with baseline values. These cutoff points were established based on the recommendations of the ASE, EACVI [5], and Brazilian Society of Cardiology (*Sociedade Brasileira de Cardiologia* - SBC) [22].

### Statistical analysis

The SPSS software version 25.0 (IBM, Armonk, NY, USA) was used for all statistical analyses. Initially, the normality and homogeneity of the variables LVEF and LV GLS were tested using the Shapiro–Wilk and Levene’s tests, respectively. Since the data showed normality and homogeneity, the one-way parametric analysis of variance test with repeated measures was used to compare the scheme every 3-weeks and 3-months. A *P* value < 0.05 was considered statistically significant.

After identifying subclinical CTX, the Chi-Square or Fisher’s test was used to compare 3-week and 3-month intervals considering both LV GLS cutoff points. A similar strategy was used to compare the 12 and 15% reference values in each regimen. In these analyses, a *P* value < 0.05 was considered significant.

Univariate Cox regression analysis followed by multivariate analysis was used to verify predisposing factors for CTX (hypertension, diabetes, dyslipidemia, smoking, age, body mass index, total anthracycline dose and use of trastuzumab). The significance level was set at *P* < 0.05.

## Results

### Participants

**Fig. 1:**
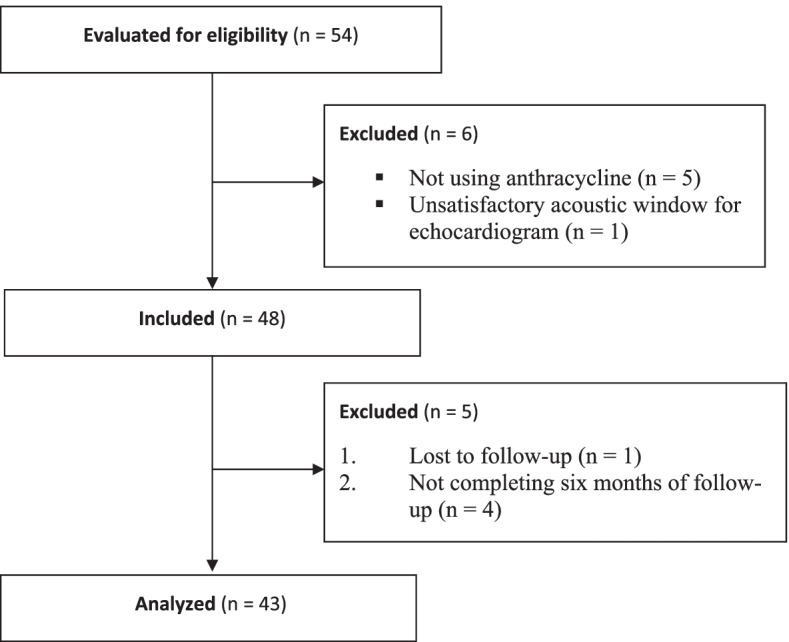
Study flowchart. n: number of patients

Initially, the study included 54 female patients. Of these, 11 were excluded (Fig. 1). Thus, data of 43 women aged between 34 and 73 years were included in the analysis (Table 1). All patients had an adequate echocardiographic window to calculate LVEF and LV GLS over the follow-up period.

**Table 1:** Demographic, clinical, and physical examination data of patients (*n* = 43)

Data	Values
**Age** (years)^a^	50.4 (8.8)
< 60 years (n/%)^b^	35 (81,4)
≥ 60 years (n/%)^b^	8 (18.6)
**BMI** (kg/m^2^)^a^	28.6 (4.8)
< 25 kg/m^2^ (n/%)^b^	7 (16.3)
≥ 25 kg/m^2^ (n/%)^b^	36 (83.4)
**BSA** (m^2^)^a^	1.70 (0.14)
**HTN** (n/%)^b^	13 (30.2)
**DM** (n/%)^b^	6 (14.0)
**DLP** (n/%)^b^	6 (14.0)
**Smoking** (n/%)^b^	5 (11.6)
**Breast affected** (n/%)^b^	
Right	22 (51.2)
Left	20 (46.5)
Bilateral	1 (2.3)
**Histological cancer subtype** (n/%)^b^	
IDC	40 (93.0)
ILC	3 (97.0)
**HER2+** (n/%)^b^	22 (51.2)
**Breast cancer stage** (n/%)^b^	
IIa	15 (34.9)
IIb	12 (27.9)
IIIa	8 (18.6)
IIIb	3 (7.0)
IV	5 (11.6)
**Total dose of doxorubicin** (mg/m^2^)^a^	248.4 (30.9)
< 250 mg/m^2^ (n/%)^b^	40 (93.0)
≥ 250 mg/m^2^ (n/%)^b^	3 (7.0)
**Trastuzumab** (n/%)^b^	18 (41.9)

*BMI* body mass index, *BSA* body surface area, *DLP* dyslipidemia, *DM* diabetes mellitus, *HER2*+ HER2 protein expression are established on immunohistochemistry, *HTN* hypertension, *IDC* infiltrating ductal carcinoma, *ILC* infiltrating lobular carcinoma

^a^Data are presented as mean (standard deviation)

^b^Data are presented as absolute frequency (percentage)

### Clinical cardiotoxicity

**Fig. 2:**
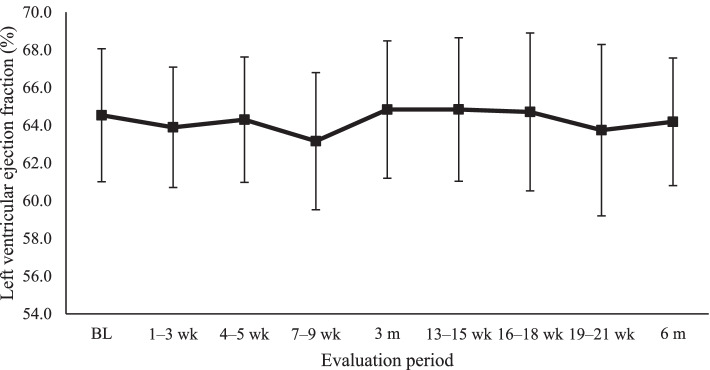
Mean left ventricular ejection fraction values every 3 weeks. BL: baseline

No patient had clinical CTX. The comparison of absolute LVEF values at baseline (64,53%), three (64,84%), and 6 months (64,19%) showed no significant differences (*P* = 0.63). A similar result was observed when comparing all evaluated times (*P* = 0.41) (Fig. 2).

### Subclinical cardiotoxicity

**Fig. 3:**
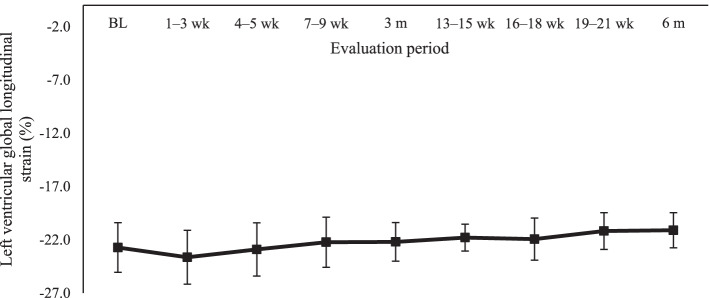
Mean left ventricular global longitudinal strain values at every 3 weeks. BL: baseline

The comparison of all absolute LV GLS values every 3 weeks showed no significant difference (*P* = 0.40) (Fig. 3). In contrast, the comparison of absolute LV GLS values at baseline (− 22,7%), three (− 22,2%), and 6 months (− 21,1%) showed a significant difference (*P* < 0.001), with the absolute value at 6 months being lower than that at baseline (*P* < 0.001) and at 3 months *(P* < 0.001). No significant differences were observed between baseline and 3-month values (*P* = 0.22) (Table 2).

**Table 2:** Percentage LV GLS variation in patients (mean and standard deviation) every 3 months

Variable	BL	1–3 wk	4–6 wk	7–9 wk	3 m	13–15 wk	16–18 wk	19–21 wk	6 m
**Mean**	**−22.7**	−23.6	−22.9	− 22.2	**−22.2**^a^	−21.8	−21.9	− 21.2	**− 21.1**^a^
**SD**	**2.3**	2.5	2.5	2.4	**1.8**	1.3	2.0	1.7	**1.6**

*BL* baseline, *LV GLS* left ventricular global longitudinal strain, *SD* standard deviation

^a^*P <* 0.05 is compared with BL

**Fig. 4:**
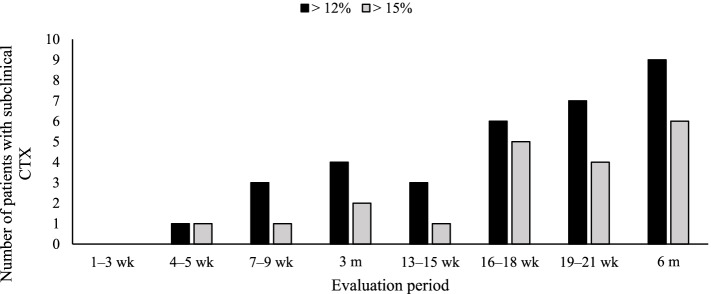
The number of patients with subclinical cardiotoxicity is evaluated constantly. Black bar: relative left ventricular global longitudinal strain (LV GLS) decrease > 12%; gray bar: relative LV GLS decrease > 15%. CTX: cardiotoxicity

**Fig. 5:**
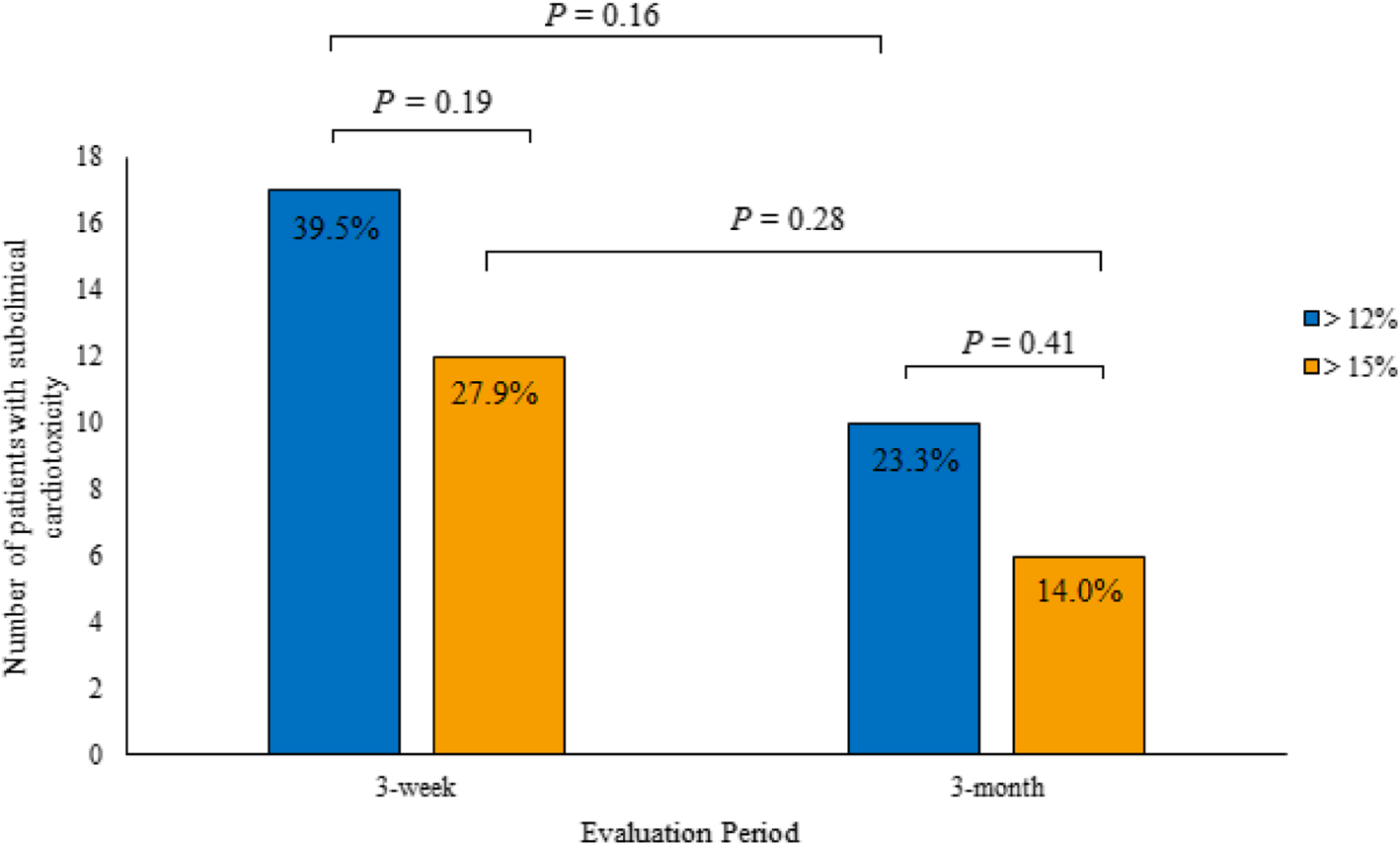
Central Illustration. Comparison of the two echocardiographic strategies for detecting subclinical cardiotoxicity. Although the absolute numbers of patients with subclinical cardiotoxicity are greater at 3-week intervals for both LV GLS variation cutoff values, the differences are not statistically significant (> 12% vs. > 15%, *P* = 0.16 vs. *P* = 0.28). Within both intervals, the numbers were not significantly different at both cutoff values (*P* = 0.19 vs. *P* = 0.41). Thus, quarterly monitoring of LV GLS may be the most appropriate echocardiographic strategy

Analysis with a cutoff point of the percentage LV GLS variation of > 15% showed that 12 (27.9%) and six (14%) patients had subclinical CTX at 3-week and 3-month intervals, respectively. However, the comparison of the two regimens showed no statistically significant difference *(P* = 0.28). After applying a cutoff point of > 12%, 17 (39.5%) and 10 (23.3%) patients had subclinical CTX at 3 weeks and 3 months, respectively. In addition, the comparison of these values showed no significant differences *(P* = 0.16) (Figs. 4 and 5).

Finally, comparing the number of patients with subclinical CTX using both reference values demonstrated no statistically significant differences in either the 3-week *(P* = 0.19) or the 3-month *(P =* 0.41) regimen. Considering these results, the Cox regression analysis considered only data from the 3-month interval regimen and a relative LV GLS decrease of > 12%. Univariate analysis revealed that age ≥ 60 years and HTN are associated with subclinical CTX. However, these associations were not confirmed in multivariate analysis (Table 3).

**Table 3:** Cox regression analysis results considering the 3-month interval and a relative LV GLS decrease of > 12%

Variable	B	SE	HR	95% CI		***P***
				Inferior	Superior	
**Univariate analysis**						
Age ≥ 60 years	− 1.322	0.556	0.267	0.090	0.793	0.018^a^
BMI ≥ 25 mg/m^2^	0.434	0.658	1.543	0.425	5.606	0.51
Doxorubicin ≥250 mg/m^2^	0.105	1.041	1.111	0.144	8.545	0.92
Trastuzumab	−0.108	0.556	0.898	0.302	2.672	0.85
HTN	−1.306	0.570	0.271	0.089	0.828	0.022^a^
DM	−0.114	0.769	0.892	0.198	4.024	0.88
DLP	−0.114	0.769	0.892	0.198	4.024	0.88
Smoking	−1.483	1.016	0.238	0.032	1.738	0.16
**Multivariate analysis**						
Age ≥ 60 years	0.732	0.713	2.080	0.514	8.418	0.31
HTN	0.883	0.731	2.4119	0.577	10.132	0.23

*B* coefficient, *BMI* body mass index, *CI* confidence interval, *DLP* dyslipidemia, *DM* diabetes mellitus, *HR* hazard ratio, *HTN* hypertension, *LV GLS* left ventricular global longitudinal strain, *SE* standard error

^a^*P* < 0.05

## Discussion

In this study, none of the 43 patients undergoing chemotherapy with doxorubicin developed clinical CTX. Although, we have identified some cases of subclinical CTX with both the cutoff points utilized (LV GLS relative reduction > 15 and > 12%). Advanced age and HTN were associated with the occurrence of subclinical CTX in univariate analysis.

Ventricular dysfunction and heart failure (HF) risks vary according to the cumulative dose of doxorubicin administered, ranging from 3 to 5% to 18–48% with doses of 400 mg/m^2^ and 700 mg/m^2^, respectively [23]. A study conducted among 2625 patients undergoing treatment with anthracyclines showed that the incidence rate of CTX was 9% during a mean follow-up period of 5.2 years. The cumulative dose of doxorubicin was 359 (± 172) mg/m^2^ in the group with CTX and 299 (± 144) mg/m^2^ in the group without CTX *(P* < 0.001) [24].

An extensive systematic review that analyzed data from 18 studies and included approximately 23,000 patients treated with anthracyclines reported an incidence rate of 17.9% for ventricular dysfunction and 6.3% for clinical HF during a mean follow-up period of 9 years. The cumulative dose of anthracyclines was consistently reported across studies as an accurate and robust predictor of CTX [25].

We identified no cases of clinical CTX, which may be due to the short follow-up period (patients were followed up for 6 months after starting chemotherapy) and/or low cumulative dose of doxorubicin administered (248.4 ± 30.9 mg/m^2^). However, there were cases of subclinical CTX (the incidence rate was 14–39.5%, depending on the criterion used).

According to the ASE and EACVI Expert Consensus for Multimodality Imaging Evaluation of Adult Patients During and After Cancer Therapy, it was suggested that a relative reduction of LV GLS > 15% (compared with baseline) is a diagnostic criterion for subclinical CTX [5]. In an Italian study analyzing strain as a guide for cardioprotective therapy in 116 patients with breast cancer, 19.8% of patients developed subclinical CTX (LV GLS reduction > 15% during a 3-month follow-up period). The prevention of clinical HF development could result from patients completing their chemotherapy after administering cardioprotective drugs (ramipril and carvedilol) [26].

The 1-year follow-up analysis of the Strain Surveillance of Chemotherapy for Improving Cardiovascular Outcomes (SUCCOUR) study has been recently published. This was the first randomized clinical trial comparing a serial analysis (every 3 months) of LV GLS vs LVEF to monitor cardioprotective therapy. Overall, 331 patients undergoing doxorubicin treatment (mean dose of 218 mg/m^2^) were analyzed. LV GLS reduction ≥12% at any time (compared with baseline) was considered an indicator of subclinical CTX. The incidence rate of subclinical CTX was 29% in the LV GLS group, whereas clinical CTX was 13.7% in the LVEF-guided group [27]. The study failed to meet the primary endpoint after the 1-year follow-up period, with no statistically significant difference in LVEF over the period evaluated (− 3.0% in the LVEF-guided group and − 2.7% in the LV GLS-guided group*, P* = 0.69). Cases of hospitalization for HF were rare (one in each group) [27]. Patients in the LV GLS group were prescribed cardioprotective therapy twice as often as those in the LVEF group. The detection of subclinical CTX resulted in interrupted cancer treatment in five patients in the LV GLS group) and two patients in the LVEF group) [27]. In addition to the higher risk of exposure to side effects and costs associated with cardiological treatment, patients in the LV GLS group had a higher risk of unfavorable cancer progression when chemotherapy was interrupted. Moreover, there were no differences in LVEF and LV GLS at the end of the 1-year follow-up period [27]. Therefore, in the absence of definitive evidence, the LV GLS should be used judiciously in the clinical follow-up of cancer patients [28]. Based on this study, the SBC adopted LV GLS change > 12% as a recommendation in the recently published Brazilian Positioning on the Use of Multimodality Imaging in Cardio-Oncology – 2021 [22]. In this study, the incidence rate of subclinical CTX observed was similar to that found in the literature. The comparison of the two LV GLS cutoffs showed no differences in the occurrence of subclinical CTX.

A systematic review and meta-analysis published in 2014 analyzed the role of myocardial strain in the early detection of CTX during and after chemotherapy. This study evaluated 1504 patients and reported that a LV GLS reduction between 10 and 15% during chemotherapy appeared to be the most useful parameter to predict the future occurrence of CTX [17]. Additionally, a systematic review and meta-analysis published by Oikonomou et al. [1] analyzed the role of LV GLS as a predictor of CTX. This analysis showed that the nine studies evaluating the percentage LV GLS variation had cutoff points that ranged from 2.3 to 15.9% (mean, 13.7%), with a sensitivity of 45 to 100% (mean, 86%) and specificity of 65 to 95% (mean, 79%) [18]. However, due to the heterogeneity of the studies analyzed and the possible existence of publication bias (limited description of the population analyzed and lack of adjustment for relevant risk factors), the meta-analysis concluded that more robust prospective studies should be conducted to define the ideal cutoff point [18].

There is still no consensus in the literature on the best cutoff point for the percentage variation of LV GLS as a predictor of CTX. This study revealed no differences in the incidence of subclinical CTX with both cutoff points, probably due to the small sample size. There were no differences in the occurrence of subclinical CTX between the two-time intervals used.

Gripp et al. analyzed the role of LV GLS as a predictor of CTX in 49 patients with breast cancer undergoing chemotherapy and identified two cases of reduced LVEF in the third month of follow-up and another three in the sixth month. The authors suggested that if the LV GLS had been analyzed after each cycle of chemotherapy (3-week interval) rather than every 3 months (routine procedure), the change in strain would have probably occurred before the decrease in LVEF. Therefore, they recommend performing echocardiography after each cycle of chemotherapy [29], which does not corroborate the findings in this study.

To the best of our knowledge, no study has directly compared the two-time intervals for monitoring subclinical CTX. Despite the limitations of sample size and the short follow-up period, more frequent monitoring does not seem to benefit the diagnosis of CTX and is associated with increased costs.

In this study, advanced age and HTN were associated with the occurrence of subclinical CTX in univariate analysis. Similarly, a systematic review and meta-analysis by Lotrionte et al. showed that in addition to the cumulative anthracycline dose, extremes of age, HTN, diabetes mellitus, extremes of weight, radiotherapy, African-American ethnicity and severe comorbidities were predictors of anthracycline-induced CTX [25].

Henry et al. observed that 4.2% of 16,456 patients with breast cancer undergoing chemotherapy developed CTX. HTN was associated with the occurrence of CTX (relative risk, 1.28; 95% confidence interval, 1.09–1.51). In addition, younger individuals (age < 50 years) were less likely to develop CTX than older ones (age ≥ 65 years) [30], thereby corroborating the findings of this study. Therefore, serial LV GLS monitoring is useful for the follow-up of patients undergoing chemotherapy with anthracyclines. Despite the small sample size and being conducted at a single center, the present study indicated that quarterly monitoring was the most appropriate for clinical practice.

### Study limitations

Intra-observer agreement was not analyzed. The low incidence of CTX in the analyzed sample was probably a result of the short follow-up period and the low cumulative dose of doxorubicin administered. Larger sample size may increase the impact of the conclusions of this study. The concomitant use of biomarkers and cardiac magnetic resonance imaging would provide additional value to the serial analysis of the strain as a predictor of CTX in suspected cases. High risk patients, specially those with cardiac structural diseases, were excluded from this analysis. This population could be the one that should be most beneficiated of the more frequent monitoring strategy. These observations should be analyzed in future studies to increase knowledge.

### Competency in patient care

Monitoring the development of subclinical CTX in patients with breast cancer who undergo treatment with doxorubicin is pivotal to the cardiovascular management of these patients. This study suggests that quarterly monitoring of LV GLS is the most appropriate echocardiographic strategy.

### Translational outlook

Further studies with larger sample sizes and extended follow-up periods are required to validate the results of the present study. Early diagnosis and treatment of CTX is necessary for improving the outcomes in female patients with breast cancer undergoing chemotherapy. Serial LV GLS monitoring is useful in this scenario. Defining the optimal cutoff point for the relative variation in the LV GLS (compared with baseline) and ideal frequency of performing serial exams are necessary measures to establish routines, avoid unnecessary expenses, and provide better care to patients with breast cancer.

## Conclusions

In this study, no patient developed clinical CTX and had a significantly reduced LVEF. The occurrence of subclinical CTX was revealed by serial measurement of the LV GLS. There was no significant difference in the incidence rates of subclinical CTX between the two LV GLS cutoff points used. More frequent echocardiographic monitoring showed no benefits compared with quarterly monitoring. Advanced age and HTN were associated with the occurrence of subclinical CTX in the analyzed population.

## Data Availability

All data generated or analyzed during this study are included in this published article.

## Abbreviations

ASE: American Society of Echocardiography
CTX: Cardiotoxicity
EACVI: European Association of Cardiovascular Imaging
HF: Heart failure
HTN: Hypertension
LVEF: Left ventricular ejection fraction
LV GLS: Left ventricular global longitudinal strain
SBC: Brazilian Society of Cardiology (*Sociedade Brasileira de Cardiologia)*
TTE: Transthoracic echocardiography
